# Lentiviral Engineered Fibroblasts Expressing Codon-Optimized *COL7A1* Restore Anchoring Fibrils in RDEB

**DOI:** 10.1038/JID.2015.364

**Published:** 2016-01

**Authors:** Christos Georgiadis, Farhatullah Syed, Anastasia Petrova, Alya Abdul-Wahab, Su M. Lwin, Farzin Farzaneh, Lucas Chan, Sumera Ghani, Roland A. Fleck, Leanne Glover, James R. McMillan, Mei Chen, Adrian J. Thrasher, John A. McGrath, Wei-Li Di, Waseem Qasim

**Affiliations:** 1 UCL Institute of Child Health, Molecular and Cellular Immunology Section & Great Ormond Street Hospital NHS Foundation Trust, London, United Kingdom; 2 St John’s Institute of Dermatology, King’s College London (Guy’s campus), London, United Kingdom; 3 Centre for Ultrastructural Imaging, King’s College London, London, United Kingdom; 4 Department of Haematological Medicine, King’s College London, The Rayne Institute, London, United Kingdom; 5 The Robin Eady National Diagnostic Epidermolysis Bullosa Laboratory, Viapath LLP, St Thomas’ Hospital, London, United Kingdom; 6 Department of Dermatology, University of Southern California, Los Angeles, California, USA

**Keywords:** AF, anchoring fibril, DEJ, dermalepidermal junction, LV, lentiviral, RDEB, recessive dystrophic epidermolysis bullosa, C7, type VII collagen

## Abstract

Cells therapies, engineered to secrete replacement proteins, are being developed to ameliorate otherwise debilitating diseases. Recessive dystrophic epidermolysis bullosa (RDEB) is caused by defects of type VII collagen, a protein essential for anchoring fibril formation at the dermal-epidermal junction. Whereas allogeneic fibroblasts injected directly into the dermis can mediate transient disease modulation, autologous gene-modified fibroblasts should evade immunological rejection and support sustained delivery of type VII collagen at the dermal-epidermal junction. We demonstrate the feasibility of such an approach using a therapeutic grade, self-inactivating-lentiviral vector, encoding codon-optimized *COL7A1*, to transduce RDEB fibroblasts under conditions suitable for clinical application. Expression and secretion of type VII collagen was confirmed with transduced cells exhibiting supranormal levels of protein expression, and ex vivo migration of fibroblasts was restored in functional assays. Gene-modified RDEB fibroblasts also deposited type VII collagen at the dermal-epidermal junction of human RDEB skin xenografts placed on NOD-*scid* IL2Rgamma^null^ recipients, with reconstruction of human epidermal structure and regeneration of anchoring fibrils at the dermal-epidermal junction. Fibroblast-mediated restoration of protein and structural defects in this RDEB model strongly supports proposed therapeutic applications in man.

## Introduction

Recessive dystrophic epidermolysis bullosa (RDEB) is a debilitating genodermatosis caused by loss-of-function mutations in *COL7A1* (Fine et al., 2014). Type VII collagen (C7) is essential for anchoring fibril (AF) formation at the dermal-epidermal junction (DEJ), and in RDEB, malformed, reduced, or absent AFs are a direct consequence of *COL7A1* mutations (Hovnanian et al., 1997). C7 is one of the main contributors of dermal-epidermal adhesion, forming “wheat-stack”-shaped, centrosymmetrically banded, semicircular loop structures known as AFs after antiparallel dimerization of two fibrils at their carboxyl (C)-termini (Burgeson et al., 1990). These can be seen extending from their amino (N)-termini that indirectly bind to hemidesmosomal α6β4 integrin via the bridging activity of laminin-332 in the lamina densa (Rousselle et al., 1997), where they protrude down to the papillary dermis encircling dermal type I and III collagen amongst other fibrous elements before terminating back in the lamina densa (Shimizu et al., 1997). Loss-of-function mutations in C7 lead to fragility of AF structures, thereby compromising the integrity of the DEJ resulting in severe sublamina densa blistering and tissue cleavage.

Clinically, skin blistering can follow even minor mechanical stress causing skin erosions from birth in many subtypes of RDEB. Moreover, chronic erosions with secondary infections that can progress to widespread, mutilating scars and joint contractures, and aggressive squamous cell carcinomas, typify the severe generalized forms of RDEB (Fine and Mellerio, 2009, Rodeck and Uitto, 2007). RDEB has a profound medical and socioeconomic impact on patients and their families (Tabolli et al., 2009). There are no curative therapies for RDEB, and supportive care, with daily dressings, meticulous wound care, nutritional support, and iron supplementation for chronic anemia are the mainstay of clinical management (Grocott et al., 2013, Mellerio et al., 2007).

Experimental therapies under development include recombinant C7 protein (Remington et al., 2008, Woodley et al., 2004, Woodley et al., 2013), infusion of allogeneic mesenchymal cells (Conget et al., 2010), hematopoietic–stem cell transplantation (Tolar and Wagner, 2012, Wagner et al., 2010), and gene therapies (Droz-Georget Lathion et al., 2015, Osborn et al., 2013, Sebastiano et al., 2014, Titeux et al., 2010). We have investigated the feasibility of ex vivo gene-modified cell-based delivery of C7 to restore AFs at the DEJ of affected skin. Although both keratinocytes and fibroblasts are involved in the production and secretion of C7, fibroblasts are generally more robust and easier to maintain in culture, making them an attractive target for such an approach (Goto et al., 2006). In addition, alternative approaches based on transduction of keratinocytes and production of engineered skin grafts may not be suitable for RDEB where the abnormal DEJ may compromise adhesion of engineered epidermal sheets. In previous studies, intradermal injections of allogeneic fibroblasts from healthy donors supported increased levels of *COL7A1* expression in patients with RDEB for several months (Nagy et al., 2011, Wong et al., 2008). However, a recent phase II double-blind randomized trial demonstrated the importance of intradermal control injections. These comprised placebo (vehicle only) reagents and resulted in similar levels of wound healing as with mismatched allogeneic fibroblasts (Venugopal et al., 2013). A significant difference between injection of vehicle and allogeneic fibroblasts was only noted at day 7 (of 28 days) in a separate trial (Petrof et al., 2013). Although the mechanism is unclear, a localized anti-inflammatory effect and upregulation of *COL7A1* from intradermal inoculation of the vehicle solution or injection needle itself (commonly used in scar remodeling) has been postulated (Nagy et al., 2011, Petrof et al., 2013, Venugopal et al., 2013). Irrespective of the mechanism, a major limitation of allogeneic injections is the immunological rejection of HLA-mismatched donor fibroblasts (Larcher and Del Río, 2015, Venugopal et al., 2013, Wong et al., 2008). An autologous approach using genetically modified RDEB fibroblasts should circumvent the risk of rejection and provide a source of locally synthesized C7. Previous reports have established the feasibility of modifying fibroblasts with a variety of vectors, including phage (Ortiz-Urda et al., 2003), gamma retrovirus (Goto et al., 2006, Titeux et al., 2010, Woodley et al., 2007), and lentivirus (Chen et al., 2002, Woodley et al., 2003), and local or systemic injection into recipient mice has provided varying degrees of evidence of restoration of skin integrity (Woodley et al., 2004, Woodley et al., 2007). We have developed a self-inactivating-lentiviral (LV) platform combined with a human phosphoglycerate kinase promoter and codon-optimized *COL7A1* for the engineering of autologous RDEB fibroblasts and have shown definitive evidence of AF reconstruction at the DEJ in a human:murine xenograft model. The production and validation of good-manufacturing-practice compliant reagents and a robust process for manufacturing engineered fibroblasts have enabled the submission of applications for regulatory approval for first-in-man testing of this therapy.

## Results

### Restoration of C7 expression in LV-*COL7A1*-transduced RDEB primary fibroblasts

**Figure 1:**
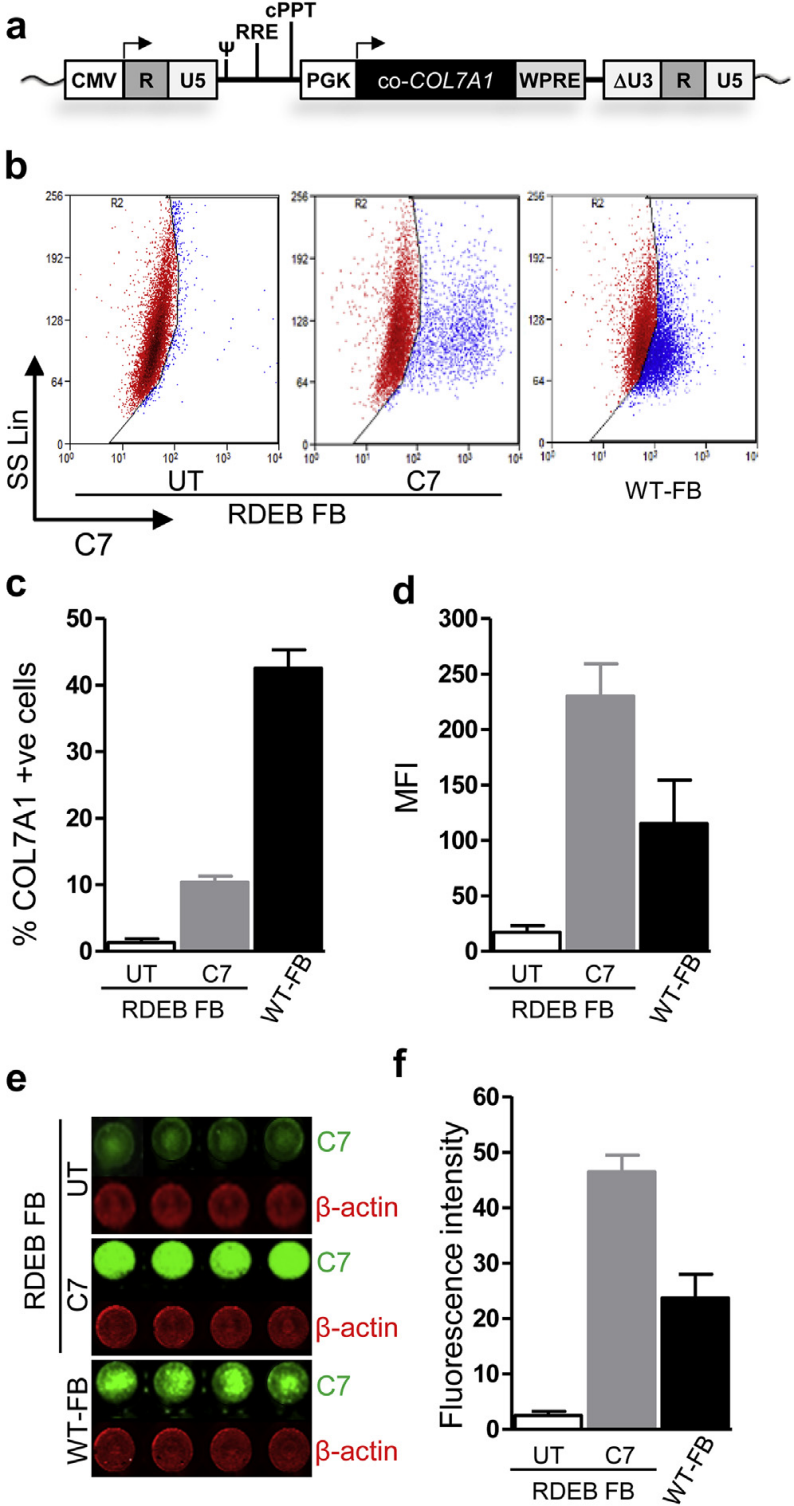
**Expression of C7 in gene-corrected RDEB fibroblasts using a SIN-LV-*COL7A1* vector.** (**a**) Configuration of pCCL-PGK-*COL7A1* lentiviral transfer plasmid shows a third-generation, split-packaging SIN vector with the deleted U3 region of the 3′LTR, internal PGK promoter, mutated woodchuck hepatitis virus posttranscriptional regulatory element (WPRE), and central polypurine tract (cPPT). Transgene *COL7A1* was codon-optimized (co-*COL7A1*) encoding the full-length *COL7A1* sequence. (**b**, **c**) Average expression of C7 in LV-*COL7*-transduced and untransduced (UT) primary RDEB-1 and -2 fibroblasts by intracellular staining and flow cytometry with corresponding mean fluorescence intensity (MFI) (**d**). (**e**) In situ expression of C7 in RDEB-1 and -2 LV-*COL7* fibroblasts using in-cell Western blotting (ICWB). Green lanes represent C7 expression; red lanes represent loading control (β-actin) expression with average immunoreactivity (**f**). LTR, long terminal repeat; LV, lentiviral; PGK, phosphoglycerate kinase; RDEB, recessive dystrophic epidermolysis bullosa; SD , standard deviation; SIN, self-inactivating. Error bars represent SD of four replicates.

**Figure 2:**
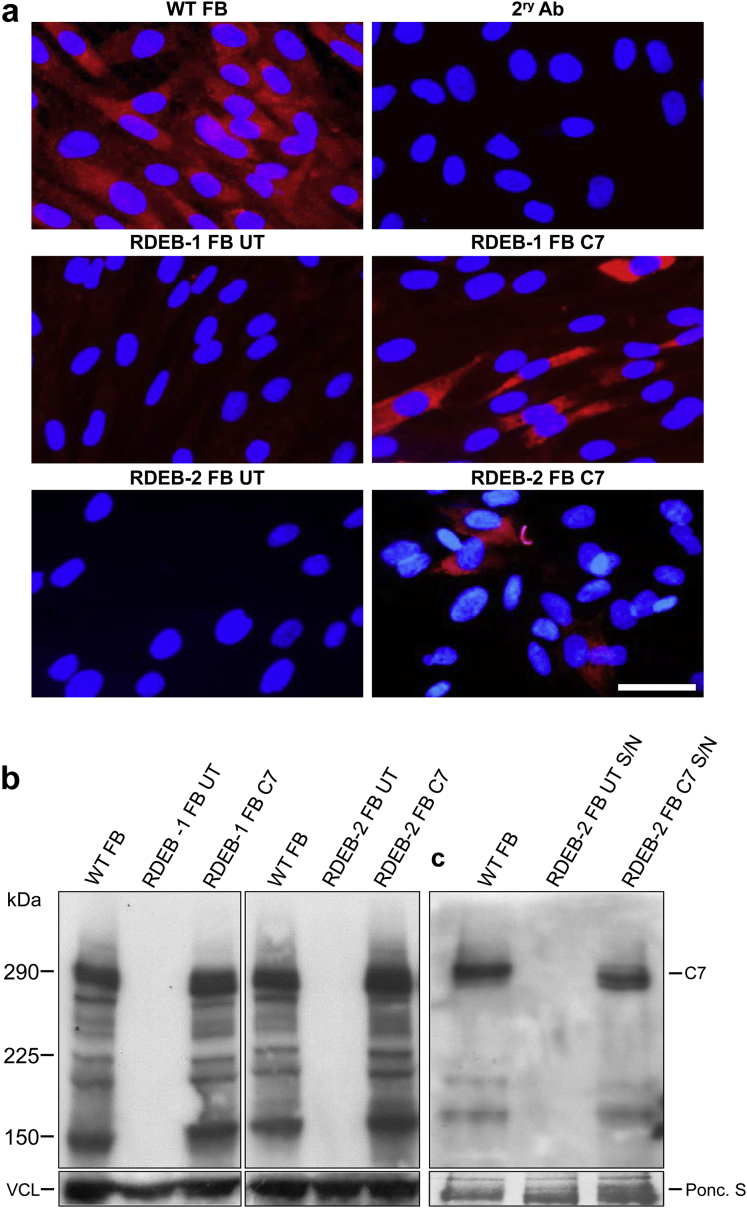
**Restoration of full-length C7 protein expression in RDEB fibroblasts.** (**a**) In situ immunocytochemistry for type VII collagen expression (C7) (red) and nuclear stain 4'.6-diamidino-2-phenylindole (blue) of either healthy primary (WT) or RDEB-1 and -2 patient untransduced (UT) or LV-*COL7* fibroblasts. C7 expression restored after LV-*COL7* transduction at MOI 5. Bar = 25 μm. (**b**) RDEB-1 and -2 fibroblast pellets were lysed before assessment by SDS-PAGE. Restoration of full-length C7 expression visualized at 290 kDa in LV-*COL7* and WT fibroblasts. The complete absence of C7 expression was seen in both RDEB-1 and -2 UT samples. Vinculin represents internal loading control. (**c**) Culture supernatant from WT, RDEB-2 UT, and LV-*COL7* fibroblasts showing secreted C7 protein at 290 kDa after lentiviral transduction. Ponceau S used as internal loading control. LV, lentiviral; MOI, multiplicity of infection; RDEB, recessive dystrophic epidermolysis bullosa; UT, untransduced; WT, wild-type.

Primary fibroblasts from patients with RDEB lacking C7 expression were transduced with a third-generation self-inactivating-LV vector encoding codon-optimized C7 (LV-*COL7*) under current good-manufacturing-practice compliant conditions using a single round of exposure at a multiplicity of infection 5 (Figure 1a). After 3 weeks of culture and expansion, flow cytometric analysis showed 9.3–12.8% of fibroblasts expressing C7 (Figure 1b–d), and this corresponded to an integrated proviral copy number of 0.12–0.14 copies/cell. In-cell Western blotting showed overexpression of C7 in transduced RDEB fibroblasts compared with untransduced and wild-type (WT) fibroblasts as measured by mean fluorescence intensity (Figure 1e and f). In situ cytostaining also detected C7 protein expression in transduced RDEB fibroblasts (Figure 2a), whereas there was no expression in untransduced RDEB fibroblasts. These results were further confirmed by Western blot analysis using a purified C7 antibody (a gift from Professor Mei Chen). Cell lysates from transduced RDEB fibroblasts revealed the expression of an approximately 290 kDa protein band corresponding to full-length C7 (Figure 2b) and expression was stable when reassessed after 8 weeks. Full-length protein was also detected in media harvested from cultured transduced RDEB fibroblasts (Figure 2c) indicating effective secretion of the recombinant protein. In view of previous reports that around a quarter of gamma retroviral vector integrants, particularly in keratinocytes, may encode truncated forms of the *COL7A1* transgene, we screened cultures for aberrant protein forms, and found that only 3 of 49 single-cell clonal populations expressed abnormally sized protein. This greatly reduced frequency was attributed to our codon optimization of the transgene, with residual low-level recombination events during reverse transcription linked to a small number of persisting repeat sequences.

### Functional recovery in LV-*COL7***-**transduced RDEB fibroblasts

LV-*COL7-*transduced fibroblasts were assessed for viability and metabolic activity using a water-soluble tetrazolium salt-1 assay, with no differences observed compared with noncorrected RDEB fibroblasts (Supplementary Figure S1 online).

**Figure 3:**
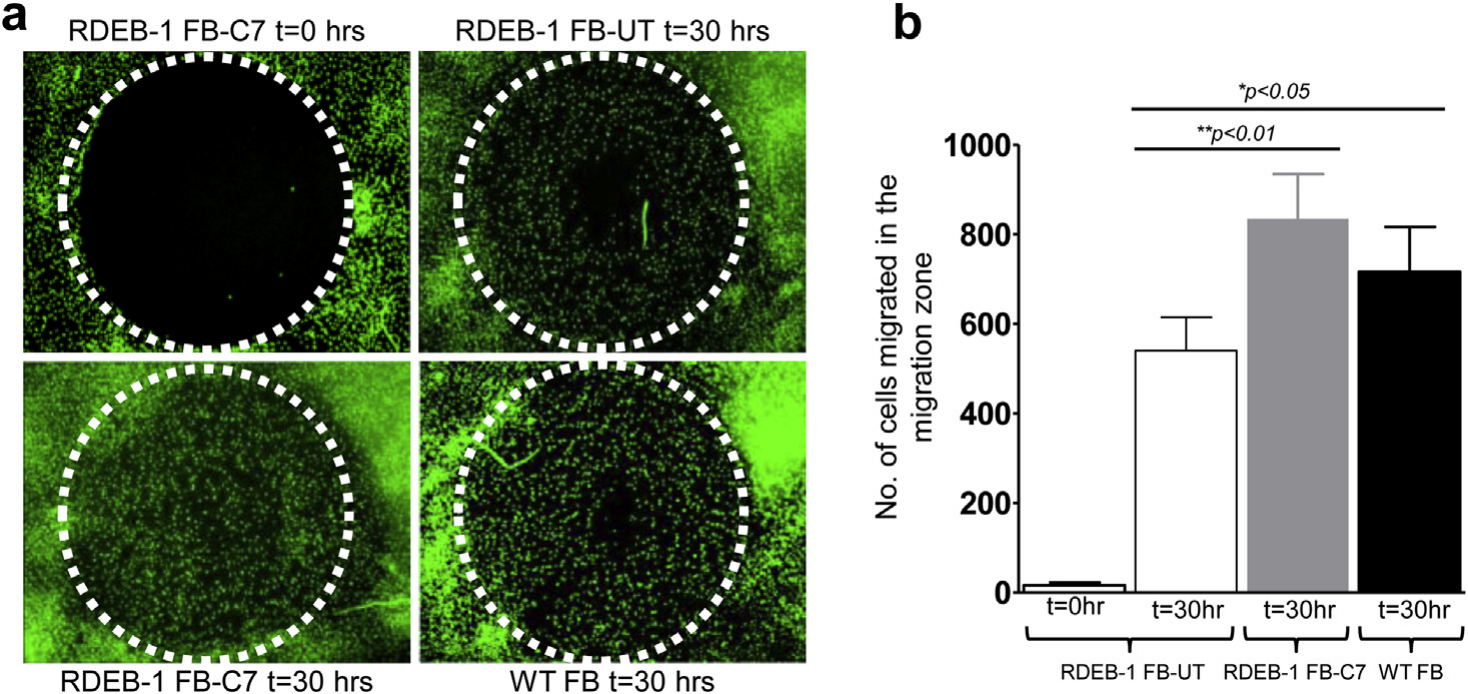
**Human RDEB fibroblasts corrected for C7 showed improved migration and “wound” closure in vitro.** (**a**) Representative micrographs of RDEB-1 fibroblasts corrected for C7 from three independent experiments show the migration pattern in a 2 mm migration zone at 30 hours (*T* = 30). (**b**) Bar graph showing normalization of migration of C7 corrected primary RDEB-1 fibroblasts toward WT values compared with uncorrected (UT) primary RDEB fibroblasts at 30 hours. Statistical analysis carried out using Student’s *t*-test. C7, type VII collagen; RDEB, recessive dystrophic epidermolysis bullosa; SD, standard deviation; WT, wild-type. Error bars represent SD of four replicates.

In migration assays, the loss of C7 in RDEB cells has been previously correlated with adverse functional effects on the kinetics of wound closure compared with WT cells. Reports have separately described both increased or decreased migration associated with loss of C7, but with normalization to WT levels after the reconstitution of C7 expression (Chen et al., 2002, Martins et al., 2009, Nystrom et al., 2013). Functional recovery in transduced RDEB fibroblasts was examined by using a two-dimensional assay of fibroblast migration across “wounds” created by cell-seeding stoppers (Syed et al., 2013). RDEB fibroblasts had reduced (*P* < 0.05) migration compared with WT fibroblasts, which was restored by transduction with LV-*COL7* (Figure 3a). The number of cells within the 2 mm migration zone was analyzed using ImageJ and revealed a significant increase in transduced compared with nontransduced RDEB fibroblasts (*P* < 0.01), with numbers similar to healthy donor fibroblasts (*P* > 0.05) (Figure 3b).

### Morphological correction of the DEJ in an RDEB human:murine skin graft model

**Figure 4:**
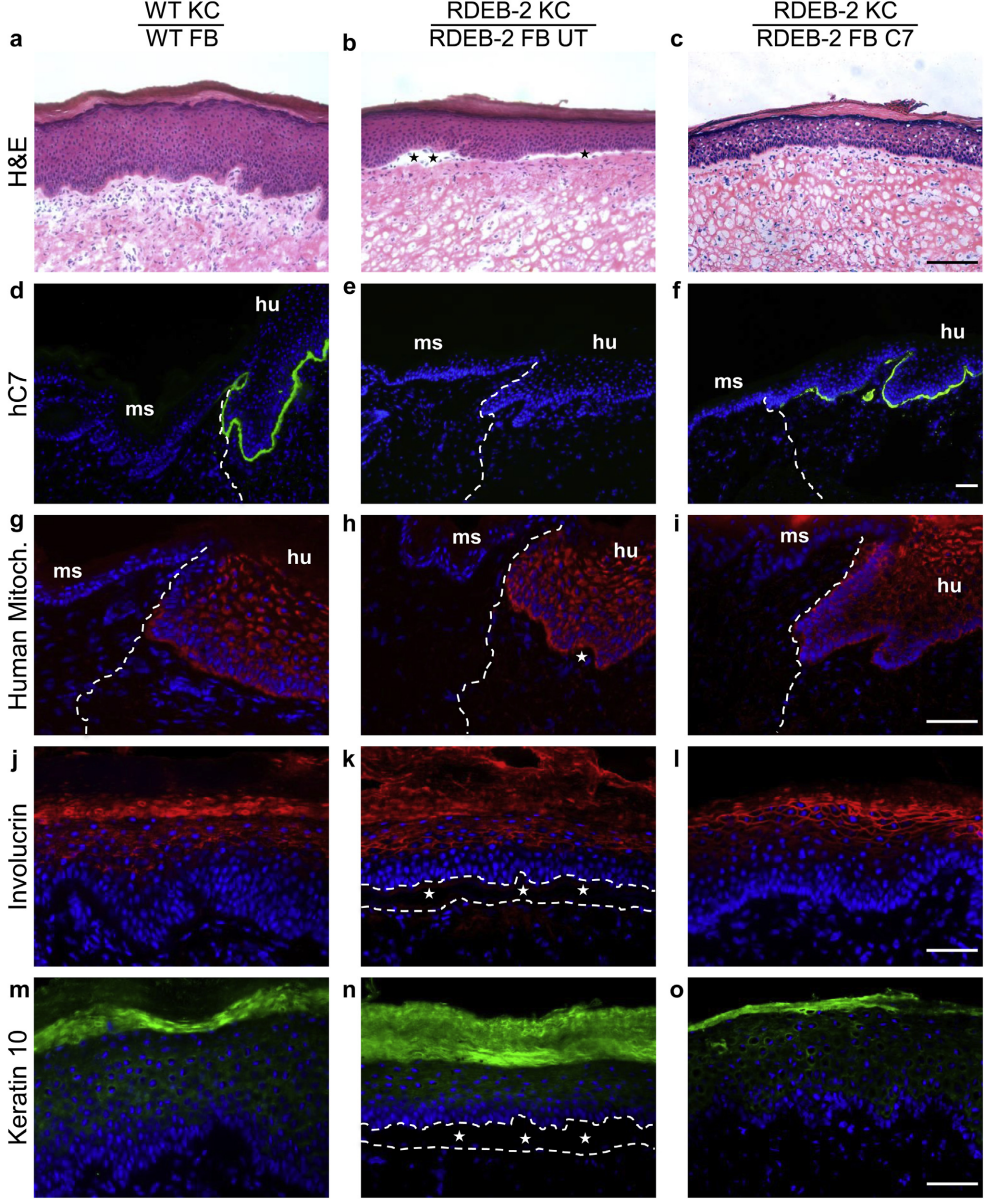
**Visualization of human origin and epidermal cytoarchitecture of bioengineered skin sheets generated on NOD-*scid* IL2Rgamma^**null**^**mice.**** (**a–c**) H&E staining of WT, RDEB-2 untransduced (UT), or LV-*COL7* fibroblast (FB) graft combination seeded with WT or RDEB-2 UT patient keratinocytes (KC). Blistering seen in RDEB-2 UT combination (stars). Bar = 50 μm. Human-specific anti-C7 antibody showing expression in healthy and LV-*COL7* grafts (**d**, **f**) but not in untreated RDEB grafts (**e**). Bar = 50 μm. (**g–i**) Human-specific mitochondrial marker identifies the human:mouse junction: the border between mouse (ms) and bioengineered human (hu) skin (dotted line). Bar = 25 μm. Involucrin staining reveals cornification (**j–l**); keratin 10 shows a later stage of KC differentiation (**m–o**). Epidermal-dermal tissue cleavage in RDEB-2 patient UT combination (dotted lines). C7, type VII collagen; H&E, hematoxylin and eosin; LV, lentiviral; RDEB, recessive dystrophic epidermolysis bullosa; WT, wild-type. Bar = 25 μm.

**Figure 5:**
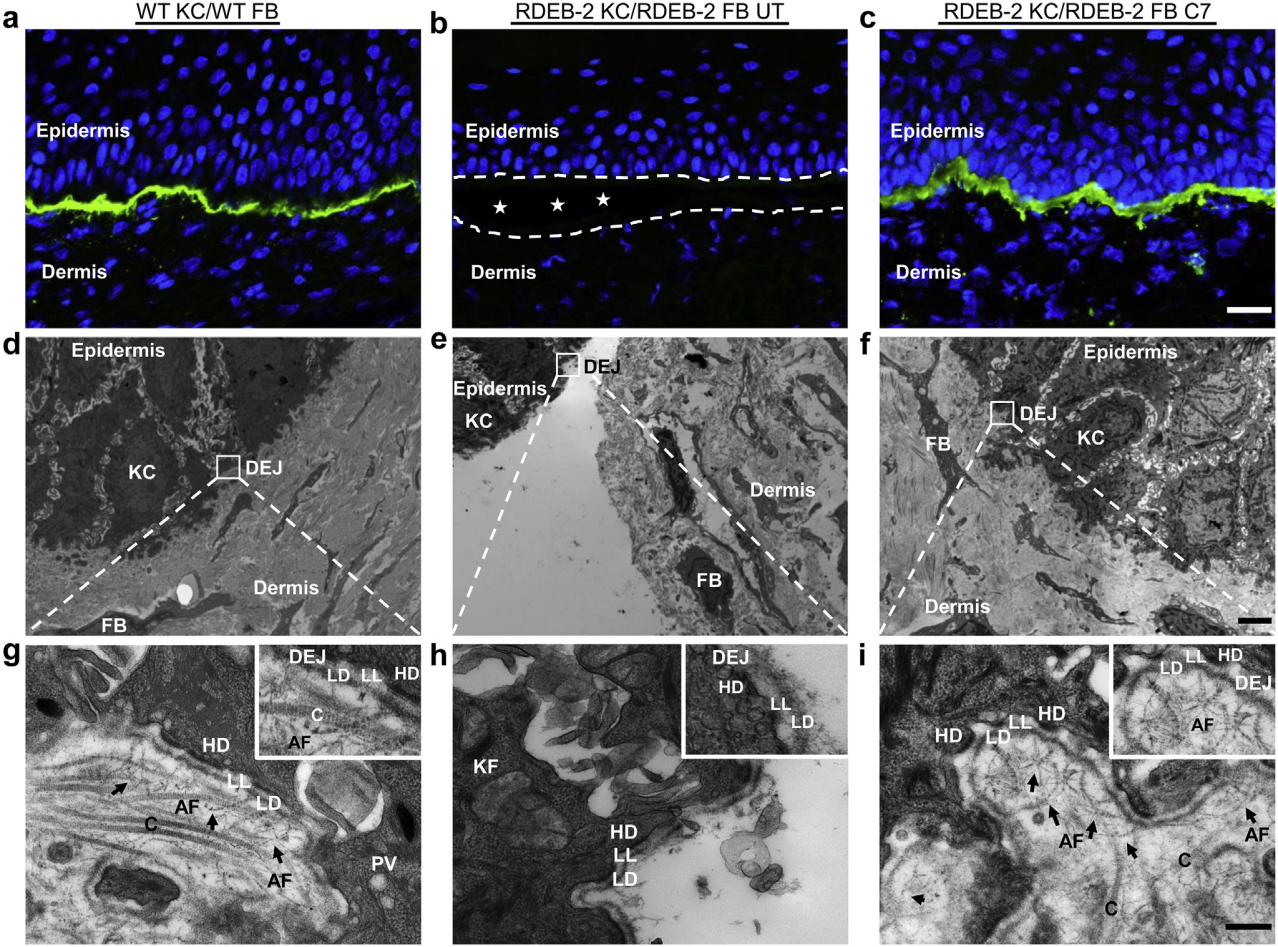
**In vivo functional correction through LV-*COL7*-mediated restoration of type VII collagen anchoring fibrils (AFs).** C7 overexpression over WT (**a**) visible in LV-*COL7* RDEB-2 fibroblast (FB) containing graft (**c**), no protein deposition seen in untransduced (UT) graft (**b**). Bar = 25 μm. TEM micrographs of WT (**d**), RDEB-2 patient UT (**e**), and LV-C*OL7* (**f**) grafts. Bar = 5 μm. (**g**) WT human keratinocyte (KC) and/or FB combination showing thick, cross-banded AFs (arrows). (**h**) Loss of AFs causes extensive tissue cleavage at the dermal-epidermal junction (DEJ) of UT RDEB-2 KC and/or FB combination with lamina densa (LD) reduplication. (**i**) UT RDEB-2 KC and/or LV-*COL7* FB combination reveals restoration of dermal-epidermal adhesion. C, collagen type I and III; C7, type VII collagen; HD, hemidesmosome; KF, keratin filament; LL, lamina lucida; LV, lentiviral; PV, plasmalemmal vesicle; RDEB, recessive dystrophic epidermolysis bullosa; TEM, transmission electron microscopy; WT, wild-type. Bar = 300 nm.

To determine whether secreted C7 produced from LV-*COL7*-transduced fibroblasts can contribute toward the deposition and incorporation of C7 into the DEJ in vivo, a modified human:murine xenograft skin model was developed using previously described procedures (Di et al., 2011, Di et al., 2012, Larcher et al., 2007). Primary RDEB fibroblasts were transduced with LV-*COL7* and seeded in a supporting fibrin matrix composed of porcine plasma and human thrombin on which primary keratinocytes were further seeded, generating a bioengineered skin graft. Control grafts carrying combinations of primary healthy or untransduced RDEB keratinocytes and fibroblasts were prepared alongside under the same conditions. The bioengineered skin grafts were grafted on NOD-*scid* IL2Rgamma^null^ mice in duplicate for each condition and allowed to mature over a period of 8 weeks. This provided an opportunity to monitor two full cycles of human keratinocyte and fibroblast development in vivo. At that point the grafts were harvested and processed for cryosectioning and transmission electron microscopy (TEM). Hematoxylin and eosin staining showed distinct and fully differentiated human epidermis with visible stratification and formation of a thick cornified layer that was readily distinguishable from murine tissue (Figure 4a–c and Supplementary Figure S2a–c online). The human derivation of the grafted area was confirmed by species-specific staining for human C7 and mitochondrial markers (complex IV subunit II) and showed clearly demarcated human:murine borders (Figure 4d–f and g–i). Human-specific staining for desmoglein further verified the human origin of the graft (Supplementary Figure S2d–e online). Epidermal proliferation and differentiation was confirmed by staining of keratin 10 and involucrin in suprabasal layers and the upper epidermal strata of terminally differentiated keratinocytes, respectively (Figure 4j–l and m–o). Taken together, these data support the adoption of a NOD-*scid* IL2Rgamma^null^ xenograft model for the reconstruction of human epidermal structures pertinent to human RDEB modeling. Severe blistering was observed in the RDEB grafts derived from untransduced fibroblasts in combination with untransduced keratinocytes and closely resembled the human disease phenotype (Figure 4b). Tissue cleavage at the junction between basal keratinocytes and the underlying dermis resulted in blister formation and epidermal sloughing upon mechanical stress. On the contrary, there was no blistering observed using the healthy donor fibroblast in combination with healthy donor keratinocytes (Figure 4a). Importantly, in grafts comprising vector-transduced RDEB fibroblasts with untransduced keratinocytes, there was also no indication of blister formation, consistent with restoration of the DEJ (Figure 4c and Supplementary Figure S2a–c) and supported by the detection of C7 expression. Robust expression of human-specific C7 was seen only in grafts incorporating transduced fibroblasts, with the deposition of the protein throughout the DEJ at levels comparable with healthy donor grafts (Figure 5a and c). C7 expression could also be detected in fibroblasts in the dermis by punctate staining in corrected RDEB grafts and healthy donor grafts, but not in untransduced RDEB cell combinations (Figure 5a–c). Collectively, these data provide compelling evidence that human C7 expression can be restored in vivo at the DEJ by RDEB fibroblasts transduced with LV-*COL7*.

### LV-*COL7***-**mediated restoration of AFs at the DEJ of RDEB skin grafts

To evaluate whether the C7 expression confirmed in grafts incorporating LV-*COL7*-transduced RDEB fibroblasts extended to the formation of AFs, ultrathin sections of each graft were imaged by TEM. The micrographs revealed an abundance sublamina densa fibrillary structures that bore the ultrastructural characteristics of normal AFs. These appeared similar to the AFs seen in healthy donor control grafts, exhibiting cross-banding and extending approximately 200 nm into the dermis, looping around type I and III dermal collagen fibers (Figure 5d, h and f, i). The morphological features of hemidesmosomes, subbasal dense plates, and anchoring filaments also resembled control skin. In addition, there was an abundance of plasmalemmal vesicles within the finger-like protrusions of the basal keratinocytes in close proximity to the basement membrane zone. In both control and transduced grafts, there was no blistering or tissue cleavage at the DEJ and a robust lamina densa throughout, consistent with the functional correction of the DEJ with restoration of dermal-epidermal adhesion by AFs (Figure 5d, h and f, i). In contrast, the nonmodified RDEB grafts had a blistering phenotype and an extensive splitting of sublamina densa leading to complete separation of the epidermis from the underlying dermis (Figure 5e and h). Moreover, the hemidesmosomes were reduced in number, smaller and, in some cases, internalized. There were no clearly discernible AFs at the DEJ, in keeping with an absence of C7 by immunofluorescent staining (Figure 5b). Overall, the data suggest that C7 secreted by a modest proportion of engineered fibroblasts is sufficient for the generation of robust AFs and the amelioration of blistering at the DEJ.

## Discussion

RDEB is a serious, painful, and disabling condition with limited therapeutic options. Based on recent experience with allogeneic fibroblasts (Wong et al., 2008), there is a strong rationale to develop a therapy for RDEB using autologous gene-engineered fibroblasts. Wong et al. reported allogeneic fibroblast cell therapy for RDEB-supported twofold increases in C7 immunostaining at the sites injected with donor fibroblasts, although it has been postulated that autocrine effects exerted on recipient keratinocytes by inflammation-induced heparin-binding EGF may also indirectly lead to increased synthesis and secretion of endogenous C7 (Nagy et al., 2011, Wong et al., 2008). An unexpected indirect upregulation of *COL7A1* was also found after intradermal injection of placebo suspension solution alone (Nagy et al., 2011, Venugopal et al., 2010), with further confirmation in a randomized clinical trial (Venugopal et al., 2010), although the precise mechanism remains unclear. In any case, such a therapy has potential advantages over gene-modified epidermal graft approaches in RDEB (Siprashvili et al., 2010), where there is concern that grafts may fail because of the nature of the DEJ defects. Localized injections of engineered fibroblasts could be used to treat troublesome blistering lesions, and systemic delivery may deliver more generalized benefit. The demonstration of safety of vector-modified cells in a localized setting would provide valuable data for subsequent systemic therapies using the same vector platform.

Whereas allogeneic fibroblasts mediated only transient benefits and were rejected over a matter of weeks, engineered autologous cells should provide longer lasting effects. This may be partly mediated through local effects triggered by the intradermal injections, but more importantly by the secretion of recombinant C7 produced in situ by a subpopulation of transduced cells. Effective fibroblast transduction has previously been reported using a variety of methods (Chen et al., 2002, Ortiz-Urda et al., 2003, Woodley et al., 2003) with a γ-retroviral delivery developed with clinical applications in mind, but troubled by low vector titer and high frequency of abnormal, shortened collagen forms (Titeux et al., 2010). Our LV platform was developed with clinical applications in mind and includes a human phosphoglycerate kinase internal promoter and co-*COL7A1* transgene with eliminated cryptic splice sites. All reagents, including sera and enzymes, were sourced for their certificates of analysis and transmissible spongiform encephalopathie compliance. Vector titer was modest, reflecting the large cargo size, and we found a greatly reduced frequency of truncated, or variant, C7 forms arising because of recombination events during reverse transcription. Our data indicate that ex vivo gene transfer to a modest number of fibroblasts using this vector results in high levels of C7 expression at the DEJ. The vector supports supranormal levels of protein expression in transduced cells, as indicated by the high intensities of C7 detected by Western blot, in-cell assays and flow cytometry. Critically, the reconstitution of C7 at the DEJ supported the regeneration of ultrastructural features including AFs.

We found that the NOD-*scid* IL2Rgamma^null^ immunodeficient mouse strain was amenable to human skin grafting without the need for irradiation or additional immunosuppression. These animals are devoid of T, B, and NK cells with additional defects of innate immunity and, thus, unable to mount effective rejection of human xenografts. Previous studies of human skin grafting (Di et al., 2011, Larcher et al., 2007) adopted the Foxn1^nu^ *nude* mouse strain, which is athymic and deficient of T cells but can retain NK and other aspects of the immune repertoire. Importantly, the grafts recovered from our model had clearly demarcated human:murine junctional boundaries, and characteristic epidermal structural features of healthy or RDEB skin, including a predisposition for epidermal detachment and blistering.

Our experiments used ex vivo transduction and graft preparation and were specifically designed to circumvent the triggering of localized paracrine effects that may be induced by injection into the epidermis. Whereas previous reports suggested that residual or baseline expression of C7 by keratinocytes may be necessary to secure a therapeutic effect (Kern et al., 2009, Wagner et al., 2010, Wong et al., 2008), we found that the restoration of C7 expression at the DEJ and AF formation was mediated by transduced fibroblasts even in combination with non-C7-expressing keratinocytes. This translated to eradication of subepidermal cleavage seen in noncorrected grafts.

With regard to future clinical translation, we have completed the production and release of a clinical batch of LV-*COL7* and demonstrated engineering of human RDEB fibroblasts under good-manufacturing-practice conditions. UK regulatory and ethics committee approval has recently been secured for a first-in-man study, designed in the first instance as a single-arm, open-label study to confirm the feasibility and safety of an approach using the localized intradermal injection of fibroblasts. If successful, comparison against control injections will follow and further systemic therapies will be envisaged using the same vector platform for the treatment of RDEB and other debilitating skin diseases.

## Materials and Methods

### RDEB skin biopsies and isolation and propagation of primary fibroblasts

A 6-mm RDEB skin biopsy was obtained with authorization from the National Research Ethics Services, Westminster (07/H0802/104), and with written informed consent from patients with RDEB-1 ((+/–) c.1732C>T p.R578X)/(+/–) c.2710+2T>C IVS20+2T.C) and RDEB-2 ((+/+) c.425A>G p.K142R). Excess connective tissue was removed using a sterile blade and the sample was incubated in neutral protease NB (1 unit/ml; SERVA Electrophoresis, Heidelberg, Germany) at 37 °C for 3 hours until the epidermis peeled off. The remaining dermis was fragmented and treated with collagenase NB6 (0.45 units/ml; SERVA Electrophoresis). The resulting cell suspension was seeded into a T25 flask and cultured at 37 °C in a 5% CO_2_ incubator.

### Production of third-generation *COL7A1*-expressing-LV vectors with human phosphoglycerate kinase promoter

pCCL is a self-inactivating-LV vector (Figure 1a) derived from HIV-1 as described previously (Dull et al., 1998). Self-inactivation was achieved through a 400 bp deletion in the 3′HIV-1 long terminal repeat and a 516 bp promoter sequence from human internal phosphoglycerate kinase promoter was included as an internal promoter (Ginn et al., 2010, Huston et al., 2011). A mutated woodchuck hepatitis virus posttranscriptional regulatory element sequence devoid of the hepadnaviral-X protein open reading frame (WPREmut6) was cloned (Marangoni et al., 2009) downstream of a full-length codon-optimized *COL7A1* transgene (Geneart, Regensburg, Germany). The vector was pseudotyped with vesicular stomatitis virus glycoprotein using a split packaging system and concentrated by ultracentrifugation. High-grade plasmids were produced, characterized, and released (PlasmidFactory, Bielefeld, Germany) for the production of good-manufacturing-practice vector stocks.

### Vector titer

The titer of concentrated LV-*COL7* virus was determined by exposing 293 T cells with serial dilutions of concentrated LV-*COL7*. Three days after transduction, cells were harvested and copies of HIV Psi packaging element (Ψ) were determined by quantitative polymerase chain reaction. Proviral integrant copy number per transduced cell was determined after normalization of Ψ with housekeeping gene albumin accounting for two albumin alleles per cell. Qualified plasmid standards encoding Ψ and human albumin sequences were used.

### Bioengineered skin preparation and grafting on immunodeficient mice

The methods for preparing and grafting bioengineered skin on immunodeficient *nude* mice have been described previously (Larcher et al., 2007). Our approach was similar, except the recipient strain was NOD-*scid* IL2Rgamma^null^. In brief, fibrinogen solution (cryoprecipitate derived from a porcine plasma source) containing 1.5 ×10^6^ WT, RDEB-2 patient ((+/+) c.425A>G, p.K142R) or RDEB-2 LV-*COL7*-transduced human dermal fibroblasts was combined with 0.025 mmol/l CaCl_2_ (Sigma-Aldrich, Gillingham, UK) and 11 IU of bovine thrombin (Sigma-Aldrich). The mixture was poured in two 35-mm wells and allowed to solidify at 37 °C for 1 hour. WT or RDEB patient human keratinocytes (1.2 × 10^6^ cells per well) were then seeded on the fibrin matrix to form the epidermal layer of the bioengineered skin. When confluent (3 days), bioengineered skins were manually detached from tissue culture wells and grafted onto immunodeficient mice. All animal procedures were performed in accordance with the United Kingdom Animals Scientific Procedures Act (1986) and associated guidelines. Grafting was performed under sterile conditions using 6-week-old female immunodeficient mice (NOD-*scid* IL2Rgamma^null^) housed under protective conditions. In brief, mice were aseptically cleansed and anesthetized, and full-thickness 35-mm-diameter circular wounds were then created on the dorsum of the mice. Bioengineered equivalents were placed orthotopically on the wound. The mouse skin removed to generate the wound was devitalized by three repeated cycles of freezing and thawing and used as a biological bandage and fixed with sutures to protect and hold the skin substitute in place during the take process. Dead mouse skin typically sloughed off within 15–20 days after grafting. Eight weeks after grafting, bioengineered human skins were harvested postmortem preserving a surrounding border of mouse epithelial tissue, snap frozen in LN_2_, embedded in optimum cutting temperature (Sakura Finetek, Alphen aan den Rijn, The Netherlands) and cryosectioned at 7 μm for histological and immunohistochemical examinations. A central portion of the human graft was placed in TEM fixative for ultrastructural imaging.

### Immunostaining of bioengineered grafted tissue

Immunofluorescence staining was performed on frozen graft tissue sections after 10-min fixation with ice-cold acetone and/or methanol (7 μm thickness). Sections were blocked for 1 hour at room temperature (RT) with 3% fetal bovine serum in phosphate buffered saline before incubation with primary antibodies against hC7 LH7.2 (Sigma-Aldrich) in a 1:500 dilution (Supplementary Table S1 online), desmoglein-1 (Fitzerald Industries, Acton, MA), involucrin (Sigma-Aldrich), keratin 10 (in-house), complex IV subunit II MTCO2 (Abcam, Cambridge, UK) overnight at 4 °C. Secondary antibody incubation with Alexa Fluor goat antimouse 488 (Invitrogen, Paisley, UK), goat antirabbit Cy3 (Life Technologies, Paisley, UK), and strep 488 was followed for 1 hour at RT. Sections were stained with 4'.6-diamidino-2-phenylindole (5 mg/ml) and mounted using a ProLong Gold antifade agent (Life Technologies). These were also stained by a hematoxylin and eosin histochemical technique. Staining was visualized and imaged using a Leica DMLB upright microscope (Leica Microsystems CMS, Wetzlar, Germany) and a Zeiss Axiophot 2 (Zeiss, Oberkochen, Germany) and processed using Image-Pro 6.2 (MediaCybernetics, Rockville, MD). Confocal imaging was carried out on a Zeiss LSM 510 Meta laser confocal microscope (Zeiss). Postprocessing was carried out using ImageJ.

### Preparation of skin grafts for TEM

For TEM, the central piece (approximately 3 × 3 mm^2^) of each skin graft was dissected out and fixed with half strength Karnovsky’s fixative (2% [v/v] paraformaldehyde, 2.5% [v/v] glutaraldehyde in 0.1 M phosphate buffer [pH 7.4]) for 3–5 hours at RT and kept at 4 °C until further processing. After the initial fixation, tissue samples were rinsed several times in phosphate buffer and postfixed with 1.3% osmium tetroxide in double distilled water for 2 hours at RT. Samples were then washed, en bloc stained with 2% uranyl acetate in 50% ethanol and dehydrated in a graded series of ethanols. Tissue samples were further equilibrated with propylene oxide before infiltration with TAAB epoxy resin, embedded, and polymerized at 70 °C for 24 hours. Ultrathin sections (70–90 nm) were prepared using a Reichert-Jung Ultracut E ultramicrotome (Eeichert-Jung, Vienna, Austria), mounted on 150 mesh copper grids (Gilder, Grantham, UK), contrasted using uranyl acetate and lead citrate and examined on a FEI Tecnai 12 (FEI, Hillsboro, OR) transmission microscope operated at 120 kV. Images were acquired with an AMT 16000M camera (Advanced Microscopy Techniques, Woburn, MA). Morphological examination and AF scoring of the TEM slides was blinded and performed by an ultrastructural microscopy specialist.

## Conflict of Interest

The authors state no conflict of interest.
