# Supplementary material for: Lentiviral Engineered Fibroblasts Expressing Codon-Optimized COL7A1 Restore Anchoring Fibrils in RDEB
Source: J Invest Dermatol. 2016 Jan;136(1):284–92. doi: 10.1038/JID.2015.364 (PMC4759620; doi:10.1038/JID.2015.364)
Supplement: Supplementary Data [file mmc1.pdf]

Supplementary methodology

Assessment of C7 expression in transduced fibroblasts using flow cytometry

Primary fibroblasts derived from subjects with RDEB were transduced with clinical grade LV-COL7 encoding lentiviral vector. Briefly, 1 x 10^5 cells were seeded in 24-well plates and transduction was carried out at MOI 5 with LV-COL7. For expression analysis, cells were fixed in Fix & Perm® Medium A (Life Technologies, Paisley, UK) for 20 mins at RT in the dark. The cells were then washed with 2-3 % FBS/PBS, in 2 ml, twice; spun down at 1,600 rpm for 4 mins at RT. The cell pellets were resuspended in Fix & Perm® Medium B with C7 antibody at a 1:25 dilution (LH7.2, Santa Cruz Biotechnologies, Heidelberg, Germany), and incubated at 4°C overnight in the dark. The cells were washed with 2 %FBS/ PBS, twice and pelleted at 16,000 rpm for 4 mins. The cell pellets were resuspended in Fix & Perm® Medium B with Alexa 488 goat or rabbit anti-mouse 2^{ry} antibody at a 1:200 dilution (Life Technologies, Paisley, UK), and incubated at RT for 1hr 30 mins in the dark. The cells were washed with 2% FBS/PBS twice, resuspended in 0.5 ml 2-3% FBS/PBS and analyzed using flow cytometry.

Cyto-immunofluorescence staining

Both LV-COL7 transduced and untransduced primary RDEB fibroblasts (minimum 1 x 10^5 cells/per coverslip in 24 well plate) were fixed in 4% neutral buffered formalin, washed once with 1x PBS and permeabilized with 0.1% Triton X-100 in PBS. Cells were incubated with blocking solution (1% BSA in PBS) for 20 mins at RT, and then incubated overnight at 4°C with C7 primary antibody at a 1:25 dilution (LH7.2, Santa Cruz Biotechnologies, Heidelberg, Germany). After washing, cells were incubated with Alexa-488 goat-anti-mouse 2^{ry} antibody at a 1:200 dilution, (Life Technologies, Paisley, UK) and DAPI (4',6-Diamidino-2-Phenylindole) (Life technologies, Paisley, UK). The slides were mounted with Prolong gold (Life technologies, Paisley, UK). Micrographs were taken using a Leica DMLB upright microscope (Leica Microsystems CMS GmbH, Wetzlar, Germany)

Immunoblotting

Transduced and untransduced cell pellets, or 48 hr cultured serum-free S/N supplemented with 50 μg/ml ascorbic acid and concentrated using ProteoExtract® Protein Precipitation kit (Merck Millipore, Hertfordshire, UK), were resuspended in cell lysis buffer composed of 50 mmol/L Tris-HCl pH8.0, 150 mmol/L NaCl, 5 mmol/L EDTA, cocktail protease inhibitors

and 1 mmol/L phenylmethanesulfonylfluoride. Samples were lyzed by pipetting the cells up and down repeatedly until the cell pellet dissolved completely, and then incubated for 20 mins on ice. The samples were then centrifuged at 12,000 rpm for 10 mins to pellet the insoluble cell debris at 4°C. The total protein concentration in the supernatant was determined using a Bio-Rad protein assay kit (BIO-RAD, Hertfordshire, UK). Samples from cell lysate were further diluted in 5x sample buffer containing 100 mmol/L dithiothreitol, 10% sodium dodecyl sulphate, 30% glycerol, 0.001% bromophenol blue, and 0.5 mmol/L Tris-HCl pH 6.8. Equal quantities of total protein were loaded in 6% sodium dodecyl sulphate polyacrylamide gel electrophoresis (SDS-PAGE). After electrophoresis, proteins were transferred to polyvinylidene fluoride membranes (PVDF) and incubated with anti-C7 antibody overnight or anti-vinculin mAb (cl.V284, Sigma-Aldrich, Dorset, UK) for 1 hr at RT with shaking. On the next day, membranes were further incubated with mouse anti-rabbit secondary antibody conjugated with HRP (Sigma-Aldrich, Dorset, UK) and signals were detected using the ECLplus system (GE Healthcare, Hatfield, UK). Total protein amounts in the samples from concentrated culture medium loaded on the SDS-PAGE were checked by Ponceau S (Sigma-Aldrich, Dorset, UK) staining of the PVDF membrane followed by protein transfer.

In-Cell Western Blotting

The protocol has been previously described (Syed *et al.*, 2011). Briefly, normal skin fibroblasts, RDEB LV-COL7 transduced and RDEB untransduced primary fibroblasts were seeded (1.5×10^4 cells/well) into each well of a 96 well plate. The cells were allowed to grow for ~30 hrs, then washed once with PBS and fixed using 4% formaldehyde for 30 mins at RT. The cells were washed 3 times using sterile PBS, permeabilized with PBS/0.1% triton X-100 and blocked in Odyssey blocking buffer (LI-COR, Cambridge, UK) for 2 hrs at RT. Cells were then incubated with mouse anti-human C7 antibody/rabbit anti-human beta actin (Abcam, Cambridge, UK) at 1:25 and 1:250 dilutions respectively overnight at 4^0C and subsequently washed with PBS/0.1% Tween-20 three times. Infrared rabbit anti-mouse IRDye 800CW (LI-COR, Cambridge, UK) and donkey anti-rabbit IRDye 700CW secondary antibody (1:500 dilution in odyssey blocking buffer) (LI-COR, Cambridge, UK) was added and the plates were incubated for 1 hr at RT. The wells were then washed with PBS/0.1% Tween-20 3 times. The plates were covered with aluminium foil and micrographs were captured on an Odyssey infrared scanner (LI-COR, Cambridge, UK) using the microplate-2 setting with sensitivity of 7 in the 800/700 nm wavelength channel.

***In-vitro* two-dimensional migration assay**

The assay was performed as described previously (Syed *et al.*, 2013; Syed *et al.*, 2012) with some modifications. Briefly, an Oris 96-well black plate (Oris migration assay kit, Cambridge Bioscience, Cambridge, UK) was used and Oris cell seeding stoppers were inserted according to the manufacturer's instructions. Serum-starved C7 transduced and untransduced RDEB primary fibroblasts were seeded at a density of 2.5 x 10^4 cells per well in each well of an Oris 96-well migration assay plates. The plate was then incubated overnight at 37°C and 5% CO_2. The next day, the cell seeding stoppers were removed and 100 ml of fresh medium was added and the plate was further incubated and the cells were allowed to migrate for ~30 hrs in the migration zone. Micrographs were captured using 4x magnification of an inverted microscope (Olympus, Japan). Cells in the migration zone were counted using ImageJ (Rasband, W.S., ImageJ, US National Institutes of Health, USA.http://imagej.nih.gov/ij/) from four independent experiments and averages plotted on graphs.

Cell viability/metabolic activity assay (WST-1)

RDEB primary fibroblasts were plated onto a 96 well plate at a density of 1.5×10^4 cells per well and allowed to grow for 24-96 hrs. After incubation water soluble tetrazolium salt-1 (WST-1) containing (4-[3-(4-iodophenyl)-2-(4-nitrophenyl)-2H-5-tetrazolio]-1,3-benzene disulfonate) assay was performed to assess cellular viability and cell metabolic activity, using the manufacturer's instructions (Roche, West Sussex, UK).

Supplementary Tables and Figures

Table S1: List of primary antibodies used in this study.

Monoclonal Ab	Fluorochrome conjugate	Target/ lineage specificity	Dilution	Supplier
**Keratin 10**	none	Suprabasal keratinocytes	1:10	In-house
**Desmoglein I**	none	Cell-cell junctions, Human specifc	1:100	Fitzgerald Industries
**Collagen type 7**	none	Anchoring fibrils Human specific	1:500	Sigma
**MTCO2**	none	Mitochondrial Complex IV subunit II	1:250	Abcam
**Involucrin**	none	Suprabasal keratinocytes	1:15,000	Sigma
**Vinculin**	none	Loading control	1:250,000	Sigma

Supplementary Figure S1

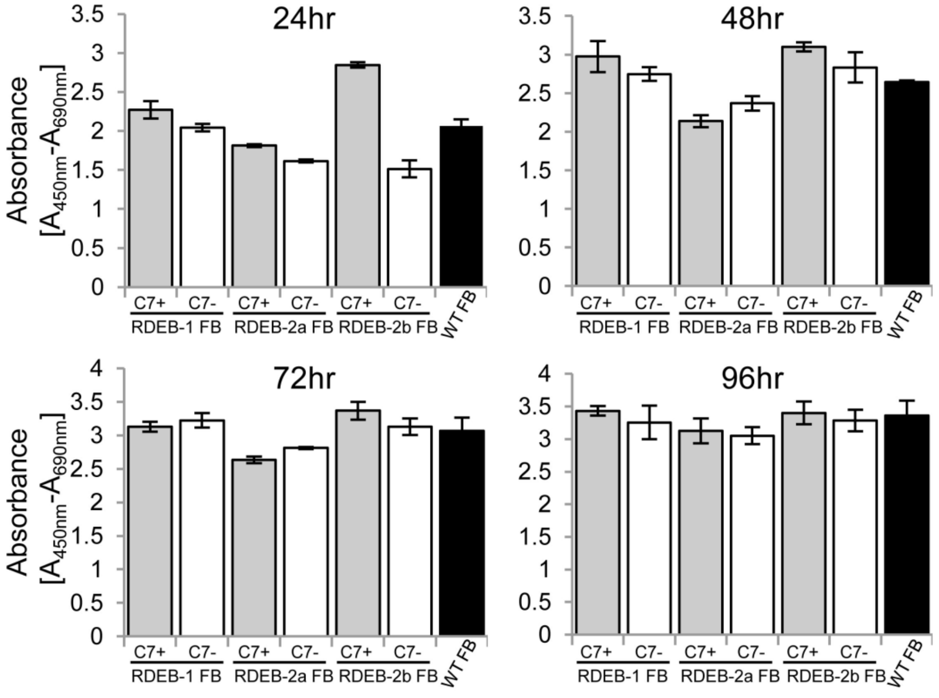

Figure S1. Metabolic activity and cell viability in cells transduced with LV-COL7 vector. (a) Transduced and untransduced RDEB fibroblasts from two subjects (RDEB-1 and two biopsies from RDEB-2 a & b) were plated in a 96 well plate ($1x10^4$ cells/well) and cultured for 24, 48, 72 and 96 hrs. Cell viability and metabolic activity using WST-1 assay was performed and results showed there were no differences between transduced and untransduced cells. Error bars represent SD of triplicates.

Supplementary Figure S2

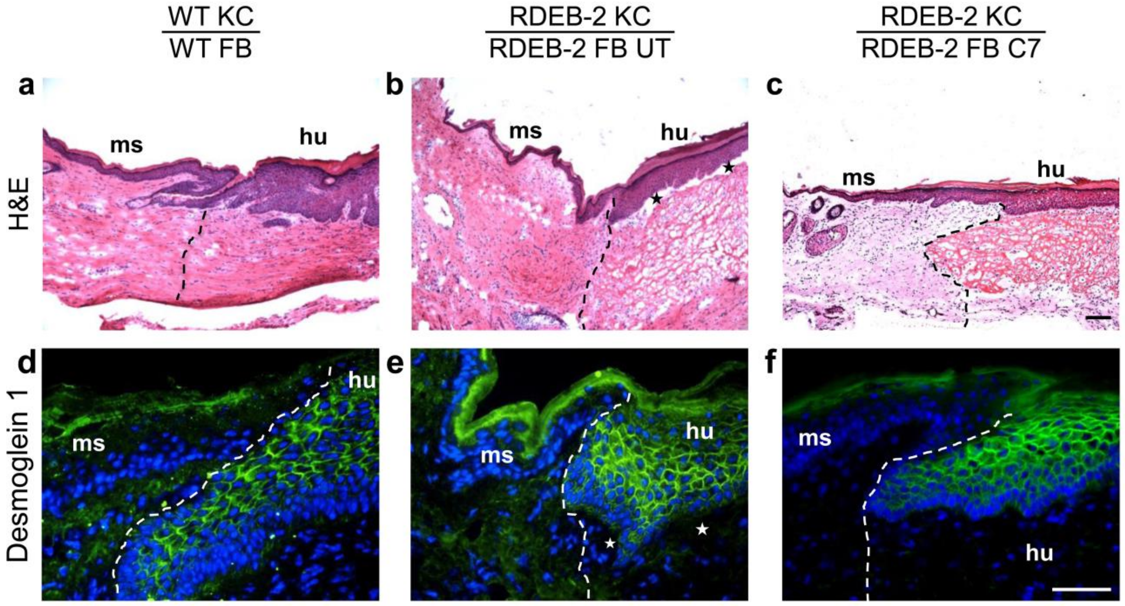

Figure S2. Identification of human-like epidermal cytoarchitecture in the bio-engineered skin sheets generated from NOD-*scid* IL2Rgamma^{null}. (a-c) Low magnification imaging of H&E stained wild type (WT), RDEB or fibroblasts (FBs) corrected graft combination showing border (dotted line) between mouse (ms) and bioengineered human (hu) skin. Bar = 50 µm. **(d-f)** Desmosomal marker desmoglein 1 was used as a human/murine border identifier. Bar = 25 µm.

Supplementary references for methodology

Syed F, Ahmadi E, Iqbal S, *et al.* (2011) Fibroblasts from the growing margin of keloid scars produce higher levels of collagen I and III compared with intralesional and extralesional sites: clinical implications for lesional site-directed therapy. *Brit J Dermatol* 164:83-96.

Syed F, Sanganee HJ, Bahl A, *et al.* (2013) Potent dual inhibitors of TORC1 and TORC2 complexes (KU-0063794 and KU-0068650) demonstrate in vitro and ex vivo anti-keloid scar activity. *J Invest Dermatol* 133:1340-50.

Syed F, Sherris D, Paus R, *et al.* (2012) Keloid disease can be inhibited by antagonizing excessive mTOR signaling with a novel dual TORC1/2 inhibitor. *Am J Pathol* 181:1642-58.
